# "The Hospital" Medical Book Supplement.—No XXIV

**Published:** 1909-12-18

**Authors:** 


### "THE HOSPITAL" MEDICAL BOOK SUPPLEMENT.—No XXIV.

CONTENTS.

| | PAGE |
|---|---|
| **Medicine—** | |
| A Textbook of Medical Treatment | 331 |
| Diseases of Metabolism and Nutrition. No. VIII., Gout | 331 |
| Psychotherapy | 331 |
| **Surgery and Anatomy—** | |
| Quain's Elements of Anatomy. Vol. III.: Neurology | 332 |
| The Medico Chirurgical Series. No. 1 The Practice of Anæsthetics | 532 |
| Manual of Surgery | 332 |
| **Miscellaneous Items—** | |
| Philanthropy and the State | 333 |
| "The Mediæval Hospitals of England" | 333 |
| The Health Resorts of Europe | 333 |
| Industrial Diseases and Accidents | 333 |
| Food and Health | 334 |
| How to Feed Children | 334 |
| Memorias do Instituto Oswaldo Cruz | 334 |
| Clinical Memoranda | 334 |
| Mosquito or Man | 334 |

### MEDICINE.

A TEXTBOOK OF MEDICAL TREATMENT. By WILLIAM CALWELL, M.A., M.D., Physician, Royal Victoria Hospital, Belfast. (London: Edward Arnold. 1910. Pp. iv. + 630. Price 16s.)

THIS textbook of medical treatment is alphabetically arranged upon very much the same lines as the already well-known Index of Treatment published in Bristol. It is well printed upon light paper, so that the volume is not at all heavy to hold, notwithstanding its bulk. In addition to the general outlines and principles of treatment of the different conditions named, there are a fair number of actual prescriptions written in full. We do not know why the author advocates 5 minims of tincture of digitalis for chronic mitral cases; our own impression is that such small doses might just as well not be given at all; benefit seldom arises from less than 15 minims thrice daily. This is an example of the way in which an apparently authoritative textbook may be quite misleading to practitioners. Were there no work obtainable upon the lines of the one before us we might think more favourably of it, for in many respects it is good; and yet it strikes one as being to too great an extent a duplicate of books already existing. Another point that we do not like about it is that it is dated 1910, when it is really published in 1909.

DISEASES OF METABOLISM AND NUTRITION; No. VIII., GOUT. By Professor Dr. H. STRAUSS, of Berlin. Translated by Dr. FOSTER, of New York. (Bristol: John Wright and Sons, Limited. London: Simpkin, Marshall, Hamilton, Kent and Co., Limited. 1909. Price 3s. 6d. net.)

THIS booklet reminds one in its shape and size of the orations that are published after being read before learned societies. Like such orations it possesses no index, which is a great drawback. Worse than some such orations, it has no lettering down the back, so that as it stands upon one's shelf one has no means of picking it out by name. The ground upon which the monographs are being published is that there is so much literature appearing upon the various subjects that it is almost impossible for any busy man to study it all and sift out that which is of practical value from the rest. Professor Strauss has succeeded in giving the gist of modern views about gout, which he deals with under the headings of (1) the differentiation of gout; (2) the pathogenesis of gout; (3) the symptoms of uricacidæmia; and (4) the therapy of gout.

PSYCHOTHERAPY. By HUGO MÜNSTERBERG, M.D., Professor of Psychology in Harvard University. (London: T. Fisher Unwin. 1909. Pp. 401. Price 8s. 6d. net.)

IT is a curious coincidence that this work should present itself for review upon the day after Dr. Savage's Harveian oration, in which he advocated strongly that the profession should seriously consider the question of hypnotism and psychology, with a view, as he said, to sift the chaff from the grain; for the book before us seems to be a genuine attempt, upon the part of a qualified medical man, who holds the most reputable post of professor of psychology in Harvard University, to sift away the chaff, and to present to the reader as candidly as may be not only the abuses, but also, and more particularly, the uses of treatment by hypnotism and suggestion. Psychotherapy consists in treating the sick by influencing their mental life. Its value in cases of neurasthenia, for example, is undoubted. The personal factor in the treatment of diseases is of great importance. The question at issue is the extent to which this factor should be used intentionally; and the author tries to indicate what is the correct and middle path between absolute scepticism or even horror on the one hand, and the opposite extreme, of which an example is afforded by some of the allies of so-called Christian Science. Almost all works upon psychotherapy written by psychologists, however, are too difficult for the average man to follow, and this applies to the work before us. Psychologists will no doubt read Dr. Münsterberg's words with relish and interest, but the average medical man needs something simpler and less wordy. The book is in three parts, the first of which deals with the psychological basis of psychotherapy; the second with the general methods of psychotherapy, its special methods and the mental and bodily symptoms which result; whilst the third deals with the place of psychotherapy in the hands of the Church, of the medical profession, and of the community respectively. We regard the subject as so very special, that we by no means endorse the author's suggestion that psychology should be an additional subject in the earlier years of the medical student's curriculum, and psychotherapy a part of the routine study in the wards. The curriculum is already overladen, and any additional subject, such as this, if studied at all generally should be left until the student is already qualified in general medicine, surgery, and midwifery.

### SURGERY AND ANATOMY.

Quain's Elements of Anatomy. Vol. III. : Neurology. By E. A. Schäfer, LL.D., F.R.S., and J. Symington, M.D., F.R.S. Part II. 11th edition. (London : Longmans, Green and Company, 39 Paternoster Row. Price 15s. net.)

The second part of the third volume of Quain's "Anatomy" is devoted to the descriptive anatomy of the peripheral nerves and organs of special sense. It is ably written and in some sections an elaborate treatise, in the editing of which many important foreign textbooks have been laid under contribution. The descriptions have been excellently done, but not better than in some other standard works on the subject, while there are few original illustrations. The edition is principally a student's manual and is by no means so suitable for the general practitioner as are the one-volume textbooks of established reputation. The advanced student, however, will find Quain a mine of information, and will be delighted with the sectional arrangement which makes it very convenient for him to take a handy sized book home with him instead of the ponderous tomes of other textbooks.

The Medico-Chirurgical Series, No. 1. The Practice of Anæsthetics, by Rowland W. Collum, L.R.C.P., M.R.C.S.; and General Surgical Technique, by H. M. W. Gray, M.B., F.R.C.S.E. Edited by James Cantlie, M.A., M.B., F.R.C.S. (London : J. Bale, Sons and Danielsson. 1909. Pp. 392. Price 10s. net.)

In the editor's preface it is explained that the purpose of the series is to deal conjointly with the medical and surgical aspects of certain branches of medical science : to intervene proctorially, as it were, and prevent that divorce of medicine and surgery which undeniably is too prevalent in textbooks. In this the first volume of the series are treated two aspects of the operative treatment of disease which are in fact not a very happy example of the union to whose rupture Mr. Cantlie objects. For, closely as efficient anæsthetisation affects the surgeon, surgical technique (in the sense in which it is here used) affects the anæsthetist not at all; a mutual understanding between surgeon and anæsthetist is very desirable, but neither should attempt to encroach on the domain of the other, and the two specialties are really not in the least advantaged by inclusion within the same covers. However, with succeeding volumes as projected, probably better instances of the importance of combined surgery and medicine in a regional text-book will be forthcoming.

To deal singly with the two component parts of the present publication, Mr. Collum has written a short textbook on anæsthetics which is, on the whole, a sound and practical monograph. It is planned throughout for the guidance of those whose experience of administration is but occasional, and is written in a pleasant style which is easy to read, if not always very correct judged as English composition. The most notable tendency, perhaps, is that towards the use of ether, which is said to be indicated for a number of conditions in which many anæsthetists hold it to be strongly contra-indicated. This is the more curious in that the open ether method receives but scant attention. Thus ether is advised for "most abdominal operations," whereas chloroform is recommended for "patients suffering from dyspnœa with cyanosis." These points are, after all, matters of opinion very largely : but we do not think the author will carry many with him when he says that profound narcosis, preferably by the ethyl chloride-ether sequence, is necessary for obstetric version and forceps operations, nor when he advises C.E. mixture for a patient in a state of collapse. There are also to be noted occasional errors which mar the reader's confidence : South Africa is not in the tropics; secondary, hemorrhage has surely nothing to do with the anæsthetist or the drugs he uses; and it is not easy to comprehend why more chloroform should be absorbed (as alleged) into the blood when it is circulating slowly through the lungs than when it is circulating fast. Notwithstanding these criticisms and occasional slipshod sentences which in some instances express the exact opposite to what the author means, the monograph is one which can be recommended to those for whom it is designed.

Turning to Mr. Gray's share of the work, we find a somewhat wide field ranged over. To sum up the antiseptic and aseptic technique of operative surgery, with after-treatment, bandaging, care of dressings, ligatures, and descriptions of emergency operations within the compass of 155 not very large pages is a task so titanic that it is not surprising to find the result somewhat scrappy. The author is rather too fond of notes of exclamation, following pyrotechnic sentences which are a little out of place in a book of this nature. He is nothing if not thoroughgoing in aseptic technique, and actually advises a prophylactic injection of 500 million staphylococci and 250 million streptococci a week before any operation on the nose, mouth, or throat. After this we read with less surprise that patients about to undergo operations on the stomach should have sterilised food the day before. In advising that a glass instrument cupboard should be kept in the hospital anæsthetising room, he has probably omitted to take into account the terror which such an article of furniture would strike into the hearts of the patients brought there; and the somewhat complicated system advocated of changing patients' clothes after anæsthetisation and before washing up seems to have several disadvantages to which sufficient consideration has not been given. He is strongly opposed to the use of gloves except for cases which are already septic and may therefore contaminate the surgeon's hands. Necessarily very incomplete as this section of the first volume of the Medico-Chirurgical series is, it contains much that should be valuable to those whose surgical education has not included a resident hospital appointment.

Manual of Surgery. By Alexis Thomson, F.R.C.S.Edin., and Alexander Miles, F.R.C.S.Edin. Two Vols. Third Edition. (Edinburgh, Glasgow, and London : Frowde, Hodder and Stoughton. 1909. 10s. net.)

We notice many changes and alterations in this third edition of "Miles and Thomson," which should make it a more valuable work than the second edition has been, admirable as the latter was. The improvement this issue shows upon the first edition is much more striking even. Miles and Thomson is essentially a students' book, but at the same time it is a valuable ally to the general practitioner in that it is eminently practical. In this issue the theoretical and purely pathological parts of the text have been cut down to the irreducible minimum; their exclusion is justified on the ground that no modern surgical textbook can deal adequately with subjects which, though formerly included in the scope of such a work, have been relegated, owing to the extensive progress that has been made during the last half-century, to the domain of special sciences. The student who desires more than an indication of the pathology should be referred to a textbook on that subject. In this edition the essentials are fully given, and they are generally accurate. The notes on surgical anatomy prefacing each chapter are concise and clear, while treatment is fully dealt with in the majority of sections. The work, as a whole, is so excellent and so carefully done that it demands a longer notice than we can give it, and we must therefore content ourselves with references to one or two points. Very many rare conditions—to instance a few, snapping hip, parosmia, Schlatter's disease, and Madelung's deformity—are described, though of necessity the space devoted to them is comparatively small, and the more important and much more common lesions are not neglected. The illustrations are judiciously chosen, and some are very good, while only a few are indifferent; and a few—fig. 187, which is certainly not a proper delineation of a Hodgen splint, for instance—are downright misleading. We are very glad to see the Bier-Klapp hyperæmic treatment so fully, and, on the whole, so excellently described, and its indications so clearly given. In the anæsthetics section spinal anæsthesia is dealt with, though we consider the authors over-estimate its dangers, as they also certainly do those of the scopolamin method. At all events, we have never had any untoward results in giving chloroform after scopolamin, and never witnessed the depression of which the authors speak. Venous anæsthesia is not dealt with, nor is the Wittek method of spinal anæsthesia—which is perhaps the best of all methods—even mentioned. We have said it is impossible to review this excellent work adequately within our available means. To epitomise our criticism in a few words: it is a good general textbook, as full as it can reasonably be expected to be, up to date, concise and clear, accurate in its teaching, and, as such books ought to be, dogmatic. It is a book which deserves to be on the practitioner's shelves, for it is one of the best condensations of modern surgical theory and practice with which we are acquainted.

### MISCELLANEOUS.

PHILANTHROPY AND THE STATE. By B. KIRKMAN GRAY. Edited by B. L. HUTCHINS. (P. S. King and Son. 7s. 6d. net.)

THERE is nothing like enthusiasm for covering faults of style and other evils most incident to writers, and in his "Philanthropy and the State" the late Mr. Kirkman Gray had not only enthusiasm but, in the social movement and its entry into nineteenth-century politics, one of the most fascinating subjects to deal with. As we read, whatever side we take in the controversy between State action and individual action, of the efforts of the early nineteenth-century "reformers," of Mrs. Fry, and the rest, we cannot avoid the feeling that the nineteenth century is far more remote than the ninth. In one of many passages, exquisite in humour, that the history of private philanthropy in the past century must describe, we come across such a defence of visiting poor people as the following: "There is charm in the visit of a superior." And the worthy writer grows quite eloquent in all sincerity on this amazing theme. Such touches Mr. Gray was careful to insert for the enlivenment of his readers; but indeed the whole subject, and at least a chapter or two of the book, abounds in them. The book is more carefully written than well written, and has an academic quality about it that may charm or repel according to the reader's taste. Still an author must be a wonderfully bad author who is dull when he has a cause to plead or a thesis to defend, and Mr. Kirkman Gray had a very definite thesis. What it was can be shortly expressed in the title which he originally chose for his book—the failure of philanthropy. He is at pains to show that every social evil, from disease to unemployment, which private philanthropy has endeavoured to tackle, it has failed to uproot, and that a study of nineteenth-century Acts of Parliament is a study of the defeat or impotence of private benevolence to do the work of social reform. Mr. Kirkman Gray died before he had completed the work, and this disarms criticism of many defects in the volume, which the thoroughly capable editing of Miss Hutchins has not corrected, though we are sure she is fully aware of them.

"THE MEDIÆVAL HOSPITALS OF ENGLAND." By ROTHA MARY CLAY. The Antiquary's Books. (Methuen and Co. Demy 8vo. 7s. 6d. net.)

IT is interesting to learn from this book, which may be regarded as a popular history of a great and little-known subject, that the voluntary principle of hospital support is a typically modern one. The period dealt with in the present volume extends to 1547, the year of Henry VIII's dissolution of the monasteries, from which Miss Clay would have us regard rightly the modern period of hospitals as starting. Mr. Kirkman Gray's "History of Philanthropy" took the same year as its starting point, and the two books might be said to form a continuous account of hospital, among other philanthropic, activities. Hospitals are ecclesiastical in origin; they were originally maisons de dieu (mesondieux), the appendages of monasteries. For by the mediæval church the virtue of charity was not left to the whim of private emotion, but enforced on all the faithful; so charity being the Church's handmaid became as much a State institution as the Church itself. The book gives a good deal of information without much real learning, and if somewhat scrappy in form is at least sensible and readable. It is also illustrated, and may be regarded as a typical member of the antiquary's series to which it belongs, for there is no doubt that the public could learn many things from its pages. It is a little difficult to see what the book has gained by the Bishop of Bristol's preface.

THE HEALTH RESORTS OF EUROPE. By THOMAS LINN, M.D. (of Nice). Edited by A. C. GLYNN GRYLLS, M.A., F.R.G.S. Seventeenth Edition. (London: Reynolds Bales' Guides, 27 Chancery Lane, W.C. 1909. 2s. 6d. net.)

WE have a high opinion of this condensed Guide, and have said so on more than one occasion when reviewing former editions. This, the seventeenth edition, is in every way worthy of whatever praise we have to give. It is full, accurate, readable, informative, and sensible—in fact, just what such a "medical and popular guide" should be. We miss a few comparatively little known resorts—Sauerbrunn, for example—but those that are given are epitomised in an ideal fashion—fares, seasons, hotels, list of doctors being concisely enumerated. Every practitioner should have the book handy for reference purposes, for it is absolutely trustworthy and fully up-to-date in its information.

INDUSTRIAL DISEASES AND ACCIDENTS. By W. J. GREER, F.R.C.S., Medical Referee under the Workmen's Compensation Acts. (Bristol: J. W. Arrowsmith. London: Simpkin, Marshall, Hamilton, Kent and Co. 1909. Price 7s. 6d. net.)

THE scope of the various Compensation Acts is now so wide, the litigation produced by them so frequent, and the points raised so debatable and so varied, that a synopsis in one volume of information on every conceivable injury or disease, about which a medical man may have to give an opinion in a court of law, is very welcome. Mr. Greer's range is certainly a wide one, and surely includes every possible morbid condition that might be made the subject of a claim. Unfortunately, in endeavouring to make his book useful to the legal profession as well as the medical he has deprived it of most of the value it might have possessed for the latter. His simplification of terms, explanations of every technicality, and assumption in his readers of ignorance of the very rudiments of anatomy and surgery result in a large proportion of his matter being useless to qualified practitioners. This and the condensation of what is really a very large subject into 300 pages prevent any but the most superficial discussion of most of the topics dealt with; and the doctor confronted with a difficult problem will scarcely find herein a sufficiently profound discussion of the factors in it to help him materially. Nevertheless, for the legal profession the book will probably be very valuable, and in any case the copious references to authors are a feature of the work to which the most emphatic praise can be afforded.

Food and Health. By Arthur E. Powell. (Methuen and Company. 3s. 6d. net.)

In the preface the author states that the object of this book is to indicate how to feed human beings correctly and healthily. Whether the book will attain its end, however, remains to be seen. It is written for the layman, and not for the professional man, and hence each subject under consideration is treated in as simple a way as possible. We confess we do not agree with much that is written in this volume. Thus, under the heading of "When to Eat," the author describes the sensations of hunger as a "sinking feeling" in the region of the stomach, though in exactly what way it sinks he does not tell us. Again, the author advocates the starting of the day's work on an empty stomach, for he believes that a man can do better work if no energy is absorbed in the digestion of food than if part of that same energy were being diverted to digesting the contents of a full stomach. The author has gone to considerable pains in consulting various references, and in quoting different authorities to bear out his facts. But whether the book is worthy of labour so expended is open to doubt.

How to Feed Children. By Louise E. Hogan. (Philadelphia: J. B. Lippincott Company. Price 5s.)

This handbook is intended as a manual for mothers, nurses, and physicians, and its first edition appeared in April 1896. Its value is testified to by the fact that it has already reached its ninth edition. The contents embody sensible suggestions as to dietaries, both for healthy infants and children, and also for those suffering from various illnesses. Allowance must be made for the fact that a few articles advocated are decidedly American, but upon the whole the dietaries discussed would apply perfectly well to any country in the temperate zone. From the practitioner's point of view the work is of value on account of the great detail with which menus for the different meals upon different days of the week are drawn up, and for similar detail in regard to dietaries for summer, for winter, for travelling, for school, and so forth. The book is very readable throughout.

Memorias do Instituto Oswaldo Cruz. (Rio de Janeiro: Manquinhos. Part I., Vol. I. April 1909.)

This is a new publication which is to appear in parts at no specified intervals, though each year at least one volume of not less than 200 pages will be issued. It consists of a number of articles, and is peculiar in that, in addition to the Spanish letterpress, each paper is also in one other language, either German, English, or French, as the case may be. The Spanish and the translated versions are printed side by side in parallel columns. Five articles are in Spanish and German, two in Spanish and French, and one in Spanish and English. If future parts contain as good material as the one before us, the publication should be a valuable one.

The articles dealt with here are upon the following subjects: The concentration of anti-diphtheritic serum by filtration; Erephopsis auricincta, a new form of fly of the family Pangoninæ, with a fine coloured illustration; anti-plague serum, with experiments upon horses; an account of a new kind of amœba, the Amœba diplomitotica, illustrated; two new species of plasmodia—Plasmodium diploglossi and Plasmodium tropiduri—being a contribution of the intraglobular parasites of the lizards, with illustrations; studies upon tuberculosis, and the staining reaction of tubercle bacilli; and a study of the biology of the Brazilian anophelides and their relation to malaria.

Clinical Memoranda. By Alexander Theodore Brand, M.D., C.M., and John Robert Keith, M.D., C.M. (Baillière, Tindal, and Cox. 3s. 6d. net.)

So far as we know the gentlemen who have here collaborated have never entered the arena of writing before, and if our surmise is correct, they are all the more to be congratulated on the excellence of this book. The title that it bears does not, perhaps, quite convey its mission. It is, in fact, a small dictionary of treatment, culled from the experience of the two writers, and most methods of treatment that they advocate are those which have been tried and tested and proved to be good. The book is divided into four parts—Medical, Surgical, Obstetrical, and Therapeutical—and under each heading are dealt with the common everyday ailments that are met with in general practice. Rare conditions are omitted, for the object is to help the busy doctor in curing the ordinary ills of life. In the Surgical division, the diagnosis and treatment of appendicitis is particularly well handled; while, on the other hand, more might with advantage have been said when the subject of fractures and dislocations was under consideration. The Obstetrical part—which, by the way, is the smallest of the four sections—is perhaps open to serious criticism. We do not entirely agree with the writers in some of their statements. Thus we doubt very much whether a case of post-partum hæmorrhage would "be instantly controlled by the inhalation of amyl nitrite," or that the best treatment for puerperal septicæmia is to introduce cotton-wool dipped in iodised phenol and "daub it all over the interior of the uterus." The remarks about forceps delivery are excellent, and will be appreciated by all general practitioners. At the end of the book we get six pages of aphorisms, many of which are very true, and could they be remembered would prove of considerable value. To the general practitioner the volume will be of service, but most of all will it prove its mission to the young doctor about to commence his life's work, for therein will he find many hints that will help him in his practice.

Mosquito or Man. By Sir Rubert Boyce, F.R.S. (London: John Murray. 1909. Pp. 267. Price 10s. 6d. net.)

This is an admirable book, which could be read with much advantage, not only by medical men, but also by any layman who is interested in the tropics. Thanks to the Schools of Tropical Medicine, immense advance has been made during the last few years in the investigation of the causes of tropical diseases and in the discovery of practical methods of preventing and even of stamping out these diseases. Sir Rubert Boyce gives the gist of our present knowledge of these things in a form which is sufficiently non-technical to be readable with interest by all.

